# Does Timing of Eruption in First Primary Tooth Correlate with that of First Permanent Tooth? A 9-years Cohort Study

**DOI:** 10.15171/joddd.2015.0016

**Published:** 2015-06-10

**Authors:** Hamidreza Poureslami, Naser Asl Aminabadi, Alireza Sighari Deljavan, Leila Erfanparast, Azin Sohrabi, Zahra Jamali, Sina Ghertasi Oskouei, Kameliya Hazem, Sajjad Shirazi

**Affiliations:** ^1^Associate Professor, Department of Pediatric Dentistry, Faculty of Dentistry, Kerman University of Medical Sciences, Kerman, Iran; ^2^Professor, Department of Paediatric Dentistry, Faculty of Dentistry, Tabriz University of Medical Science, Tabriz, Iran; ^3^Faculty of Dentistry, Tabriz University of Medical Science, Tabriz, Iran; ^4^Assistant Professor, Department of Paediatric Dentistry, Faculty of Dentistry, Tabriz University of Medical Science, Tabriz, Iran; ^5^Assistant Professor, Department of Oral Science, Faculty of Dentistry, Tabriz University of Medical Science, Tabriz, East Azerbaijan, Iran; ^6^Faculty of Medical Science, Tabriz University of Medical Science, Tabriz, Iran; ^7^7Student Research Committee dentistry, Faculty of Dentistry, Tabriz University of Medical Science, Tabriz, Iran

**Keywords:** Deciduous, dentition, permanent, tooth eruption

## Abstract

***Background and aims.*** Predicting the teeth eruption time is a valuable tool in pediatric dentistry since it can affects scheduling dental and orthodontic treatments. This study investigated the relationship between the eruption time of first primary and permanent teeth and the variation in the eruption time considering socioeconomic status (SES) in a 9-year population- based cohort study.

***Materials and methods***. 307 subjects were examined at bimonthly intervals during the first and second years of life and then at six-month intervals until the eruption of first permanent tooth. Eruption times of primary and permanent tooth were recorded for each child. A modified form of Kuppuswamy’s scale was used to assess the SES.

***Results.*** Among 267 subjects completed all follow-ups, the eruption time for first primary and permanent teeth indicated a direct strong correlation; in that one month delayed or early eruption of firstprimary tooth resulted in 4.21 months delayed or early eruption of first appearing permanent tooth (r = 0.91, n = 267, P <0.001). No significant correlation was observed between the eruption time of first primary and first permanent teeth and SES (P = 0.67, P = 0.75, respectively).

***Conclusion.*** The eruption timing for the first primary tooth had a correlation with the first permanent tooth eruption tim-ing, while SES did not have any influence on eruption times.

## Introduction

The chronology and sequence of eruption of human primary and permanent teeth are important milestones during a child’s development. Estimation of eruption schedule is a very valuable tool in child’s dental health planning including diagnostic, preventive and therapeutic measures in pediatric dentistry and orthodontics.^1^ Information on tooth emergence is also the key indicator of maturity in the diagnosis of certain growth disturbances, and in estimating the chronological age of children with unknown birth records in forensic dentistry.^2^ Moreover, the prediction of teeth eruption times is useful in interceptive guidance of occlusion, especially to determine eventual extractions of deciduous teeth and timing of orthodontic treatment.^3^ Of further note, information on the timing and sequence of human tooth emergence is also valuable when analyzing human growth and development, predicting the age of individuals and for understanding the effects of genetic and environmental influences on growth processes.^4^ On the other hand, variations in the timing of the eruption are a major concern for parents. Therefore, the specific times of teeth emergence provide an important resource for general dental practitioners, orthodontists and pediatric dentists.^2^

The normal eruption of deciduous and permanent teeth into the oral cavity occurs over a broad chronologic age range.^5^ Genetic, hormonal factors, gender, ethnic, nutrition and growth parameters, craniofacial morphology, body height and weight have been proposed as determining factors in normal eruption. Furthermore malformations, premature loss of primary teeth, traumatic injuries, malocclusions and some diseases may modify the rate of tooth eruption.^5^

The relationships between time and order of eruption of primary and permanent dentitions have received little attention. Nanda found a relation with borderline significance between the times of eruption of the two dentitions in boys.^6^ Although, according to Lysell et al,^7^ knowledge of eruption times of primary teeth apparently does not allow any general predictions about the permanent ones, but they suggested a clinically demonstrable relationship between the times of eruption of the second primary molars and first permanent molars. A prospective longitudinal study in Swedish children showed that the correlations between the primary teeth and the permanent successors were weaker than the correlations within the primary dentition, and the permanent dentition.^8^

In addition, it has been shown that children from higher socioeconomic backgrounds show earlier tooth emergence than children from lower socioeconomic classes,^9^ while others do not support this theory.^10^ It is thought that better health care and nutritional factors influence earlier tooth development and emergence.^2,10^

Sparse and inconsistent data exist on the relationship between the eruption time of primary and permanent dentition. Moreover, based on the current evidence, moderating or modulating the effect of socioeconomic status on the time of tooth eruption is still controversial. Therefore, the present population-based cohort study was designed to determine whether there is a relationship between the eruption time of the first erupting primary tooth and the first erupting permanent tooth during a 9-year period. Moreover, in the present study, we aimed to explore the effect of variation in the time of tooth emergence considering socioeconomic status (SES). The following two specific questions were intended to test our hypotheses: Is there a relationship between the eruptions time of the first primary and permanent teeth? Is there a relationship between the eruptions time of the first primary/permanent teeth and SES?

## Materials and Methods

### Samples

The participants of this longitudinal study included infants attending the urban Health Care Homes for regular assessments from April to September 2003. The Health Care Homes serve as care centre for both pregnant women and children in a given area, and therefore, the referring patients can be assumed a good sample of the general population. Children were selected considering the following inclusion criteria: full-term healthy infants, born from uncomplicated pregnancies and deliveries; birth weight 2500 grams or more; complete physical and mental health and no confounding medical history. 307 infants who matched the inclusion criteria were referred to the Department of Paediatric Dentistry at Tabriz University of Medical Sciences for comprehensive dental examinations. Once admitted, the children were examined by a post-graduate student under supervision of two experienced paediatric dentists. Infants whose parents were willing to take part in the study were consecutively included after explaining the nature of the study and signing a written informed consent form. The study design which was in accordance with the Helsinki Declaration of Human Rights was submitted to and approved by the Committee for Ethics in Research on Humans at Tabriz University of Medical Sciences (Ref number: 6428).

### Assessing Tooth Emergence

The study ran from April 2003 to October 2012. During the study course, a total of four calibrated examiners performed the examinations. All subjects were examined at bimonthly intervals during the first and second years of life and then at six-month intervals until the eruption of the first permanent tooth. Oral examinations were done using a dental mirror under good illumination. Careful observation and palpation of the alveolar ridges were done buccally and lingually to evaluate the characteristic bulge of a tooth. At each visit, the timing of primary and permanent teeth eruption were recorded on the chart. A tooth was considered erupted when any part of its crown had penetrated the gingiva and was visible in the oral cavity^1^. Parents were instructed to record the date of tooth eruption on a specially designed dental chart, kept in the child’s health book and regularly brought to the Department of Pediatric Dentistry. Patients’ charts in the department were updated in the subsequent visit. The parents were also advised to bring back their child if they noticed any signs of tooth eruption between the scheduled times. Additionally, the subjects were monitored for any systemic or local conditions which could adversely affect tooth development and eruption during each visit.

### Assessment of Socioeconomic Status (SES)

The most widely accepted scale for urban populations has been proposed by Kuppuswamy in India in 1976. We introduced a modification to the Kuppuswamy’s scale for use in the Iranian population, which takes into account the national price indices in Iran (Table 1). In the modified scale, the educational and occupational criteria remain the same because of similar educational and occupational milieu. Initially to modify the economic criteria, the family income per month for each group, which is stated in Indian Rupees (INR) in the original scale, was converted to Iranian Rials (IRR). The modified scale can be administered very quickly in any setting for large community surveys as well as small scale studies, and it has provision of updating the scale over the years to maintain its high validity. This will make the scale relevant and useful and also allow individual researchers to modify it according to the period of their research. We prepared this questionnaire and asked all parents to complete it at the first dental visit of their child.^11^

**Table 1:** Socioeconomic status (SES) questionnaire modified for Iranians

**Category**	**Score**
**Education**	
Professional or Honors	7
Graduate or Post-graduate	6
Intermediate or Post-High School Diploma	5
High School Certificate	4
Middle School Certificate	3
Primary School or Literate	2
Illiterate	1
**Occupation**	
Profession	10
Semi-Profession	6
Clerical, Shop-owner, Farmer	5
Skilled worker	4
Semi-skilled worker	3
Unskilled worker	2
Unemployed	1
Family Income per Month (in Rial)^*^	
≥20000000	12
10000000-19999999	10
7500000-9999999	6
5000000-7499999	4
3000000-4999999	3
1000001-2999999	2
≤1000000	1
**Socioeconomic Class based on Total Score**	
Upper	26-29
Middle	11-25
Lower	<10

Kappa was used to assess the inter-rater reliability. To this end, in 50 cases selected randomly, examinations were completed by two paediatric dentists. The agreement between their estimations about the eruption times in study samples was calculated using kappa coefficient. The variable was assessed two-sided. The agreement obtained between examiners was excellent (0.92). Intra-class correlation coefficient (ICC) was also used in this study to assess the reliability of the quantitative variables. In cases in which there was a need for assessing the reliability of the observer him/herself, test-retest was done in first 20 samples for the observers.^12^

### Statistical Analysis

The data were analyzed using the SPSS software (version 17). Descriptive statistics including means ± standard deviations and frequency (%) were calculated for all variables. The main statistical assessment addressing the research question was chi-square test to compare data. The correlations between variables were assessed using the Pearson’s correlation coefficient test. Multiple regression analysis was used to assess the association between the predictor variables and outcomes. The means of groups were compared by one-way ANOVA. The Tukey test was used for two by two group comparisons and the Kappa statistic was calculated for inter-rater reliability assessment. Q-Q plot and Kolmogorov-Smirnov test showed a Normal distribution of data. P < 0.05 was considered statistically significant.

## Results

From a total of 307 children (159 boys and 148 girls) initially enrolled in the study, 267 participants (141 boys and 126 girls) completed the follow-up assessments.

In this study, the agreement between the examiners was excellent (Kappa=0.92, P < 0.001)‏.

The time of eruption of first primary tooth was significantly earlier in girls than in boys (P = 0.02), while this value was not significant between genders for first permanent tooth (P = 0.11).

The eruption time for the first primary tooth was 8.5±3.2 months for boys and 6.9 ± 2.9 months for girls (overall average, 7.8 ± 3.2 months; Min, 3; Max, 15). First primary teeth to erupt were, in a respective order, mandibular central incisors (83.1%), maxillary central incisors (13.5 %) and mandibular lateral incisors (3.4 %; Table 2).

**Table 2:** The number (%) of first emerging primary and permanent tooth in the studied population (n = 267)

**Gender**	**First erupted primary tooth**			**P value**	**First erupted permanent tooth**		**P value**
	**Mandibular**	**Maxillary central**	**Mandibular**		**Mandibular first**	**Mandibular first**	
	**central incisor**	**incisor**	**lateral incisor**		**molar**	**incisor**
**Boy**	117	18	6			
	(82.9)	(12.7)	(4.2)		93 (65.9)	48 (34.0)	
**Girl**	108	15	3				
	(85.7)	(11.9)	(2.3)	0.67	81 (64.2)	45 (35.7)	0.77

The eruption time for first permanent tooth was 87.9 ± 15.2 months for boys and 82.7 ± 15.6‏ month for girls (overall average, 85.4 ± 15.5 months; Min, 53; Max, 122). In permanent dentition, the first teeth to erupt were mandibular first molars (65.2%) followed by the mandibular central incisors (34.8%).

**Figure 1.**
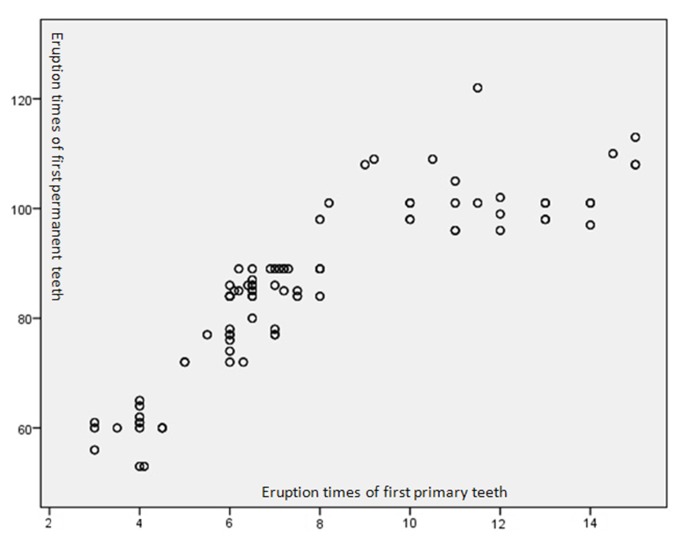
Chart showing the correlation between the times of eruption of first primary and first permanent teeth. (R^2^ = 0.82)

The findings of the present study revealed that variations in the eruption time of permanent teeth as consequence of deviations in the eruption time of primary teeth expands on a longer time span. The eruption time for first primary and permanent teeth indicated a direct strong correlation, so that one month delayed or early eruption of first primary tooth resulted in 4.21 months delayed or early eruption of first appearing permanent tooth (r = 0.91, n = 267, P < 0.001; Figure 1). Based on the results, a formula for predicting of the eruption time of first permanent was derived as:

Eruption time of first permanent tooth = (eruption time of first primary tooth × 4.21) + 52.61 months.

Also the mean eruption time of first primary and permanent teeth in each socioeconomic level (upper, middle and lower) was assessed in this study and the results are given in Table 3. There was no significant correlation between the eruption time of first primary and first permanent teeth and SES (P = 0.67, P = 0.75, respectively).

**Table 3:** Eruption times of first primary/permanent teeth and socioeconomic status (SES)

**SES**	**N**	**First eruption timing**	
		**Primary**	**Permanent**
**Lower**	56	8.1±3.7	83.9±15.1
**Medium**	178	7.8±3.2	85.4±15.5
**Upper**	33	7.0±2.2	88.4±17.4
**P value**		0.67	0.75

## Discussion

Tooth eruption is an important event during child’s development, and significant deviations from accepted norms of eruption times are often observed in clinical practice, which are a source of concern for parents. Therefore, the present study attempted to investigate the relationship between eruption times of the first appearing primary and permanent teeth, and thereby, provide a prediction tool for eruption time of permanent teeth. In the present research, we also intended to explore the relationship between variations in timing of tooth emergence and socioeconomic status.

The present study provides conclusive evidence linking the eruption time of the first appearing primary tooth and the first permanent tooth. The delay in the eruption of primary teeth resulted in an increased delay in the eruption of permanent teeth. Interestingly, one month delay in the eruption of primary tooth extends to a 4.21 months delay in the eruption of first permanent tooth. Therefore, based on the results of the present study, one could cautiously conclude that knowledge of the eruption time of first appearing primary tooth allows general prediction of the eruption time of first permanent tooth.

There are several lines of evidence that explain different degrees of correlations between the eruption times of primary and permanent dentitions. Several underlying mechanisms could explain the observed relationship in eruption times of primary and permanent teeth. Both genetic and environmental factors acting during odontogenesis are associated with tooth eruption.^3,7^ Molecular studies indicate a complex interplay of regulatory genes, leading to a cascade of signaling molecules that determine eruption rates;^13^ however, the nature of the links between the genome and phenotypic variation remains unknown.^14^ Hatton^15^ concluded that the majority of variations in the timing of tooth eruption resulted from genetic, rather than environmental, factors. The previous studies have shown four loci associated with timing of permanent teeth eruption and two loci with timing of primary teeth eruption.^3^ Human growth hormone has been also suggested to influence the periodontal ligament.^16^ Tooth eruption is also regulated by some cytokines, including epidermal growth factor and transforming growth factor beta, interleukin-1, colony-stimulating factor-1, and eicosanoids.^17^

In addition, the clear correlation between eruption times of primary and permanent teeth in the present study could be attributed to a number of interacting environmental factors. Among these factors affecting tooth eruption are race—perhaps because of hormonal, nutritional and genetic differences, environment, climate, and socioeconomic factors.^18,19^ Contrary to our finding that socioeconomic factors did not demonstrate a causal effect on eruption time both in primary and permanent teeth, a significant relationship between tooth eruption and socioeconomic status has been reported.^2^ It has been suggested that children from higher socioeconomic class get better health care and nutrition which influence earlier development of dentition.^2^ Agarwal et al^20^ reported that chronic malnutrition extending beyond the early childhood is correlated with delayed teeth eruption and most of the teeth showed a one-to-four-month variation from the mean eruption time. The high metabolic demand of the growing tissues might influence the eruptive process.^21^ This is additional confirmation to the systemic and environmental cause of tooth eruption.^22^ However, some studies, consistent with our finding, did not support this theory.^10,23,24^

Nutritional status and socioeconomic influences, for example, would seem unlikely to be the same as in other countries, and these are thought to be important influences.^25^ The difference between the impacts of socioeconomic condition on teeth emergence in dissimilar studies may be attributed to the difference in nutritional status in various countries.

Furthermore, normal variations of the eruption time have been shown to be determined by a general factor such as gender. Contrary to previous research,^26,27^ our results suggest that the eruption of primary teeth occurs significantly earlier in girls than in boys. Gender is reported to have either no effect on the timing of teeth emergence or a minimal but significant effect in favor of boys or girls.^1,26,28^ With regards to the permanent teeth, the present study showed no significant gender-related difference in the eruption times, which is in line with previous literature indicating no significant difference between eruption times of first permanent molar in girls and boys.^25^ This earlier‏ eruption of permanent teeth in girls described in some studies is thought to be attributed to earlier‏ onset of teeth maturation;^25^ but generally, the weight of current evidence provides fairly weak support for the hypothesis of gender-mediated differences in tooth eruption, and therefore, no single pattern can properly characterize gender differences in the pattern and timing of tooth eruption both in primary and permanent teeth worldwide.^1^ However, most studies support the hypothesis of Demirijian & Levesque^29^on a developmental crossover in which males lead females in the anterior dentition and females lead males for the posterior dentition.

More interestingly, variations in the eruption time of primary incisors were extended to almost 14 times the variation in the eruption of permanent incisors. This phenomenon can also be included in the discussion of general factors considered to affect the normal variations in the eruption time. The eruption of permanent teeth expands on a longer period of time, between 6 and 13 years, being submitted to individual variations that are more often and more complex than in the case of deciduous dentition,^30^ which usually emerges within the first 2.5 years of life. Our results suggest that one month delay or expedition in eruption of first primary teeth can cause 4.21 months delay or expedition in eruption of first permanent teeth, indicating a direct strong correlation between the eruption times of first primary and first permanent teeth. Since the variation in tooth eruption is believed to be multi factorial with factors capable of affecting both dentitions, the presence of a correlation in the eruption time of the primary and permanent dentitions can be justified.

There are limitations to the present study. The eruption time is described as the moment the tooth pierces the gingiva/keratinized mucosa. This is actually the “time of emergence.” A disadvantage of this method is that the exact time of emergence is hard to determine. Premature loss or extraction of primary teeth because of trauma or dental caries can influence the time of emergence of permanent teeth and is suggested to be considered in future studies. In addition, determining tooth emergence is further dependent on the timing of observation and, when determined longitudinally, it is dependent on the time span between observations. Although patients were visited bimonthly and parents were instructed to record the date of tooth eruption on a specially designed dental chart to reduce the bias, a great compliance on the parents’ side is needed, which can be difficult to achieve.

Although current findings have highlighted the link between the eruption time of the first appearing primary and the first permanent tooth, such conclusions should be weighed carefully considering the fact that this relationship may be mediated through various genetic and environmental intervening factors. Therefore, additional work is warranted in embedding the relationship between eruption time of the primary and permanent teeth within any given socioeconomic class, nutritional status, hormonal factor and racial context.

## Acknowledgements

This study was supported by grants from Tabriz University of Medical Sciences. The authors would like to thank the staff at the Departments of Pediatric Dentistry for their assistance.
